# The role of the amygdala in the perception of positive emotions: an “intensity detector”

**DOI:** 10.3389/fnbeh.2015.00178

**Published:** 2015-07-07

**Authors:** Louise Bonnet, Alexandre Comte, Laurent Tatu, Jean-Louis Millot, Thierry Moulin, Elisabeth Medeiros de Bustos

**Affiliations:** ^1^Department of Neurology, Besancon University HospitalBesancon, France; ^2^Department of Research in Functional Imaging, CIC 808, Besancon University HospitalBesancon, France; ^3^Laboratory of Integrative and Clinical Neuroscience, EA 481, SFR FED 4234 UFC-CHRU-EFSBesancon, France

**Keywords:** amygdala, emotion, IAPS, fMRI, electrodermal response, emotional intensity

## Abstract

The specific role of the amygdala remains controversial even though the development of functional imaging techniques has established its implication in the emotional process. The aim of this study was to highlight the sensitivity of the amygdala to emotional intensity (arousal). We conducted an analysis of the modulation of amygdala activation according to variation in emotional intensity via an fMRI event-related protocol. Monitoring of electrodermal activity, a marker of psychophysiological emotional perception and a reflection of the activation of the autonomic nervous system, was carried out concurrently. Eighteen subjects (10 men; aged from 22 to 29 years) looked at emotionally positive photographs. We demonstrated that the left and right amygdalae were sensitive to changes in emotional intensity, activating more in response to stimuli with higher intensity. Furthermore, electrodermal responses were more frequent for the most intense stimuli, demonstrating the concomitant activation of the autonomic nervous system. These results highlight the sensitivity of the amygdala to the intensity of positively valenced visual stimuli, and in conjunction with results in the literature on negative emotions, reinforce the role of the amygdala in the perception of intensity.

## Introduction

One of the main challenges of neuroscience in the field of emotions is the modeling of anatomical structure and its functional response underlying emotional experience. The two main models of emotion, categorical, and dimensional, are each underpinned by neural and psychophysiological patterns consistent with and validated by numerous studies (Anderson et al., 2003a; Dolcos et al., 2004; Lewis et al., 2007; Vytal and Hamann, 2010). According to discrete emotion theory, each primary emotion has a constant and specific neural and psychophysiological pattern. The dimensional model involves attractive and repulsive systems. It defines emotions by two parameters: their valence (pleasant to unpleasant or positive to negative) and their intensity, also defined as arousal (calm to excited).

The amygdala is an anatomical and functional crossroads in the emotional process of which the role has been studied by both the categorical and dimensional models of emotions. Initially, the amygdala was considered to be “the organ of fear” according to a categorical model (LeDoux, 2003). Functional neuroimaging studies (mainly PET) then expanded the role of the amygdala to the recognition of unpleasant negative emotions (Morris et al., 1996; Lane et al., 1997; Paradiso et al., 1999). However, the development in functional imaging techniques has demonstrated that the amygdala may not only be specific to unpleasant stimuli. Indeed, the amygdala showed an activation in response to both pleasant and unpleasant stimuli (Anderson et al., 2003b; Murphy et al., 2003; Small et al., 2003; Wager et al., 2003; Zald, 2003; Winston et al., 2005; Costafreda et al., 2008; Sergerie et al., 2008; Ball et al., 2009; Costa et al., 2010; Morrison and Salzman, 2010; Cunningham and Kirkland, 2014). Additionally, a meta-analysis has highlighted the lack of specificity of the amygdala to a specific primary emotion (Sergerie et al., 2008). These results might be a reflection of the failure of the categorical model.

The dimensional model now seems more appropriate in studying the role of the amygdala in emotion. The challenge was then to decide if the amygdala encodes information about valence or intensity of emotion. First, it has been shown that the amygdala is activated in correlation with the intensity of negative stimuli (Canli et al., 2000; Taylor et al., 2000). Then, studies have expanded the role of the amygdala to the perception of emotional intensity, irrespective of valence, in the chemosensory field, with olfactory and gustatory stimuli and with semantic stimuli (Anderson and Sobel, 2003; Anderson et al., 2003b; Small et al., 2003; Cunningham et al., 2004; Phan et al., 2004; Lewis et al., 2007; Costa et al., 2010). It is easier to control the variation of intensity regardless of valence in the chemosensory field rather than with visual stimuli, enabling the perception of intensity to be studied without bias related to variations in valence. Winston et al. demonstrated amygdala sensitivity to the intensity of olfactory stimuli with an extreme valence, but not to the intensity of stimuli of average valence (Winston et al., 2005). This suggests that the sensitivity of the amygdala to variations of emotional intensity might be dependent on valence. This explains why some authors have indicated that the amygdala might be a detector of emotional valence (Anders et al., 2004, 2008).

In regard to visual emotional stimulation, the most widely used stimuli are images from the International Affective Picture System (IAPS). Each of these images is provided with a normalized value of valence (*from 1, very pleasant to 9, very unpleasant, with 5, neutral*) and intensity (*from 1, very calm to 9, very excited*). Now there is a link between valence and intensity. The more intense images are often the most pleasant or unpleasant. It follows that, in the field of visual emotional stimulation, it is difficult to vary the intensity regardless of the valence, which may cause bias in studies, leading to raising doubt about the sensitivity of the amygdala concerning levels of emotional intensity (Lane et al., 1997; Anders et al., 2004, 2008) and about whether the amygdala encodes intensity or valence (Anders et al., 2008). However, it has been shown that increased amygdala activity is associated with greater arousal. This effect has been reported both when pleasant and unpleasant pictures were analyzed together and when just negative stimuli were analyzed (Lane et al., 1999; Garavan et al., 2001; Zald, 2003). Moreover, some studies use neutral stimuli as references and base states to compare amygdala activation in response to positive and negative visual emotional stimuli. The amygdala is also activated, although more weakly, in response to “neutral” stimuli (valence around 5), such as faces or photographs (Taylor et al., 2000; Sergerie et al., 2008). So there could be some confusion in these studies between low intensity and neutrality of the valence (i.e., neither pleasant nor unpleasant) which is actually an average valence (valence around 5). To avoid bias it would be preferable to study positive and negative stimuli separately when using visual emotional stimuli from the IAPS. Numerous studies highlighted the role of the amygdala in the perception of the degree of emotional intensity in many other domains (Anderson and Sobel, 2003; Anderson et al., 2003b; Small et al., 2003; Cunningham et al., 2004; Phan et al., 2004; Costa et al., 2010). Demonstrating the sensitivity of the amygdala to variations in intensity of positively valenced pictures would enhance previous results in the literature in favor of the amygdala encoding intensity of emotions.

The subject's perception of the two dimensions of valence and intensity in emotional feeling can be evaluated by self-scoring. Furthermore, emotions can be objectively evaluated by monitoring the autonomic nervous system (Lang et al., 1993; Anders et al., 2004; Kreibig, 2010). Indeed, during the emotional process, the autonomic nervous system is activated. Electrodermal activity is a psychometric marker, indicating activation of the autonomic nervous system. It has been validated that its variations are correlated with the emotional intensity felt (Dawson et al., 2007). However, when used in fMRI studies, we can question whether the electrodermal reaction is the actual result of the emotional stimulation or a reaction to the noisy and stressful environment of the MRI scan.

The aim of our study was to demonstrate the role of the amygdala in the perception of the intensity of pleasant emotions when using positively-valenced images without using negative emotional stimuli (unpleasant). In the MRI scanner, we simultaneously recorded electrodermal activity (EDA) in order to indicate the emotional intensity perceived by the subject. In order to control the effect of our stimuli, we replicated our protocol of emotional stimulation outside the scanner 1 month later to evaluate the effect of our stimuli, without the effect of the scanner. Our hypothesis is that increasing the intensity of positive visual emotional stimuli increases amygdala activation. This result should complement knowledge regarding negative as well as positive emotions in other domains (using olfactory, auditory, gustatory or semantic stimuli). We could then consider the fact that the human amygdala encodes the intensity of emotional stimuli whether positive or negative.

## Methods

### Participants

Eighteen healthy participants (10 men, all right handed, mean age 25 years, range 22–29) with normal or corrected-to-normal vision, and no history of neurological and psychiatric disorders, participated in this study. Written informed consent, as well as a safety-screening questionnaire to undergo magnetic resonance imaging (MRI), was obtained from each participant. The study received the approval of an Institutional Review Board (CPP *Est II*).

### Stimuli

Stimuli were 75 positively valenced (IAPS ratings >4.6; mean = 5.90 ± 0.49) colored visual stimuli selected from the IAPS (Lang et al., 2008) (see Table 1 for a full description). We selected three groups of 25 images each, stratified according to their intensity based on the IAPS norms (Lang et al., 2008) (mean intensity ratings ± SD; low intensity = 2.77 ± 0.17; moderate intensity = 4.53 ± 0.30; and high intensity = 6.35 ± 0.3). We carefully matched these subsets with respect to semantic content, human faces and human figures, animals, and scenes. In addition, we ensured that IAPS valence ratings did not statistically differ between each group (Kruskall-Wallis test, *p* = 0.13). The mean luminance as well as the contrast of images were controlled. Every image was transformed so that the mean luminance of every group was equal. Mean luminance for the group of low intensity (respectively medium intensity, high intensity) was 87.0 (respectively 87.2, 89.5). As there were very little differences with the contrast (mean contrast (±SD) for the 75 images = 0.115 ± 0.003), no change was made for this parameter. No statistical difference was observed between groups for neither the “luminance” parameter, nor the contrast. From these 75 pictures, we planned to create three new groups of stimuli, according to the intensity ratings made by our subjects in this study. The analyses in this study were based on these three new groups of stimuli.

**Table 1:** **Visual stimuli from the International Affective Picture Systems (IAPS)**.

**Description**	**IAPS number**	**Valmn**	**Aromn**
Lizard	1121	5, 79	4, 83
Leopard	1310	4, 6	6
Gannet	1450	6, 37	2, 83
Jaguar	1650	6, 65	6, 23
Octopus	1947	5, 85	4, 35
Woman	2025	5, 78	4, 3
Cheerleaders	2034	5, 9	4, 93
NeuWoman	2038	5, 09	2, 94
Girl	2320	6, 17	2, 9
ThreeMen	2370	7, 14	2, 9
Girl	2411	5, 07	2, 86
ManW/Dog	2521	5, 78	4, 1
Chess	2580	5, 71	2, 79
Beer	2600	5, 84	4, 16
Dance	2606	5, 92	4, 78
Dancer	2616	5, 97	4, 96
Woman	2620	5, 93	2, 72
Shadow	2880	5, 18	2, 96
Gold	3005, 2	5, 98	4, 84
EroticFemale	4232	5, 95	6, 28
EroticMale	4490	6, 27	6, 06
EroticMale	4503	6	4, 93
EroticMale	4531	5, 81	4, 28
EroticMale	4538	5, 91	4, 65
EroticCouple	4604	5, 98	6, 09
EroticCouple	4651	6, 32	6, 34
EroticCouple	4669	5, 97	6, 11
EroticCouple	4692	5, 87	6, 39
EroticCouple	4693	6, 16	6, 57
EroticCouple	4697	6, 22	6, 62
Flower	5000	7, 08	2, 67
Flower	5020	6, 32	2, 63
Flower	5030	6, 51	2, 74
Boat	5390	5, 59	2, 88
Cockpit	5455	5, 79	4, 56
Mushroom	5520	5, 33	2, 95
Mushroom	5530	5, 38	2, 87
HangGlider	5626	6, 71	6, 1
Cave	5661	5, 96	4, 15
Farmland	5720	6, 31	2, 79
Grain	5726	6, 23	2, 84
Flowers	5731	5, 39	2, 74
Leaves	5800	6, 36	2, 51
Desert	5900	5, 93	4, 38
Volcano	5920	5, 16	6, 23
Lightning	5950	5, 99	6, 79
Picnic Table	7026	5, 38	2, 63
Fork	7080	5, 27	2, 32
Headlight	7095	5, 99	4, 21
Fire Hydrant	7100	5, 24	2, 89
Bus	7140	5, 5	2, 92
Teeth	7195	6, 02	4, 54
Scarves	7205	5, 56	2, 93
Pizza	7351	5, 82	4, 25
Sushi	7477	6, 12	4, 82
Window	7490	5, 52	2, 42
Street	7496	5, 92	4, 84
Card Dealer	7503	5, 77	4, 21
Stairs	7504	5, 67	4, 25
Skyscraper	7510	6, 05	4, 52
Jet	7620	5, 78	4, 92
Skyscraper	7640	5	6, 03
City	7650	6, 62	6, 15
Violin	7900	6, 5	2, 6
Hiker	8158	6, 53	6, 49
Rock Climber	8160	5, 07	6, 97
Cliffdiver	8178	6, 5	6, 82
Bungee	8179	6, 48	6, 99
Ice Climber	8191	6, 07	6, 19
Volcano Skier	8192	5, 52	6, 03
Surfers	8206	6, 43	6, 41
Motorcycle	8251	6, 16	6, 05
Wingwalker	8341	6, 25	6, 4
Biking/train	8475	4, 85	6, 52
Woman	8620	6, 04	4, 6

### Experimental procedure

This study was composed of two sessions. In the first session, we used functional magnetic resonance brain imaging (fMRI) to examine regional brain activity during a spontaneous emotion reactivity task. Our marker of autonomic activation was EDA, monitored in the scanner. We chose a passive task in order to avoid potential inhibition of amygdala activity by the prefrontal cortex because task instructions involving a form of attentional processing reduce the likelihood of amygdala activation compared to the passive processing of emotional stimuli (Hariri et al., 2003; Keightley et al., 2003; Taylor et al., 2003; Costafreda et al., 2008). Prior to the experimental session, subjects were familiarized with the stimuli by viewing 10 pictures from the IAPS, which were not included in the experiment. Participants were instructed to experience any feelings or thoughts the pictures might elicit in them. In the scanner, subjects saw pictures on a screen, positioned at the head of the scanner, via binoculars positioned on the head coil. Stimuli were applied using an event related design. Each picture was presented for 2 s. Stimulus presentation was jittered in time, with an inter-trial interval varying from 8 to 12 s (mean = 10 s). During the inter-trial interval, a black fixation cross on a white background remained on the screen and was our baseline condition. All stimuli were presented in a randomized order. Inter-trial interval was also randomized in order to maintain the subject's attention. The time interval between two events was chosen to allow the recording of peripheral physiological responses (Breska et al., 2011) as well as to allow blood flow to return to homeostatic levels. The task lasted about 15 min.

In the second phase, 1 month later, the same participants performed the same paradigm, but outside the scanner. After this second session, all stimuli were presented in a second pass where subjects rated how arousing they had experienced each stimulus on a scale ranging from non-arousing (intensity value of 1) to arousing (intensity value of 9) on a paper-and-pencil version of the self-assessment-manikin (Bradley and Lang, 1994). This session allowed us to compare skin conductance responses (SCRs) with visual emotional stimuli inside and outside the MR scanner to evaluate the effect of MRI on SCR. Moreover, it provided us with the personal ratings of emotional intensity felt by our participants for each stimulus. Verbal recordings of experienced intensity after scanning have been shown to reliably represent emotional experiences during scanning (Phan et al., 2004) and avoid biases introduced by monitoring one's own emotion during scanning (Taylor et al., 2003; Phan et al., 2004).

### SCR acquisitions and analysis

Skin conductance was recorded using an MP-150 psychophysiological monitoring system (BioPac Systems, Santa Barbara, CA). We used Ag/AgCl electrodes filled with isotonic NaCl unibase electrolytes attached to the volar surface of the second phalanx of the second and third fingers of the left hand (non-dominant hand) (Fowles et al., 1981). Amplification utilized a constant voltage technique to measure absolute conductance. Electrodermal activity (EDA) was acquired at a sampling rate of 1000 Hz. The skin conductance signal was recorded simultaneously with functional imaging, time-locked to visual stimuli via an analogic system between Biopac and the computer using E-prime. The acquisition of EDA during the second session 1 month later was identical.

The MP-150 module performed analog-to-digital conversion of the amplified signals and passed data to a computer running Acknowledge 4.2 software (BioPac Systems, Santa Barbara, CA) for analysis of waveforms.

The SCR data were processed using high-pass filter and smoothing to remove scanner-induced artifacts. The tonic component was then extracted from the phasic component, in order to suppress the effect of the precedent stimulus on the SCR of the next one (Lim et al., 1997). We defined SCR as an increase of more than 0.02 microsiemens of the skin conductance level, occurring between 1 and 6 s after presentation of the stimulus (Dawson et al., 2007).

For each stimulus we calculated the frequency of SCRs (percentage of SCR among the 18 subjects), the magnitude (mean value computed across all stimulus presentations including those without a measurable response) and the amplitude (mean value computed across only those trials on which a measurable response occurred) of SCRs across all the subjects (Dawson et al., 2007). SCR amplitude measurements were logarithmically transformed. For the SCR magnitude measurements, a one is added to all SCR amplitudes before a logarithmic transformation in order to normalize data.

Correlations between both sessions (inside and outside the scanner) were calculated for frequencies as well as magnitudes.

### Imaging acquisition parameters

Imaging data was collected at Besancon University Hospital using a 3-Tesla (General Electric Healthcare Signa H.D. Milwaukee, WI, USA) MR system with a standard 40 mT/m gradient using blood–oxygen level-dependent (BOLD) fMRI. Foam cushions were used to minimize head movements within the coil. Functional MRI runs were acquired parallel to the anterior-posterior commissural line, covering the entire cerebrum using an echo planar imaging (EPI) sequence: echo time (TE) = 35 ms, flip angle (FA) = 90°, matrix size = 128 * 128, field of view (FOV) = 256 mm, slice thickness = 4.5 mm, 30 slices and repetition time (TR) = 2500 ms. Before the first run, a high-resolution, T1-weighted, three-dimensional data set encompassing the whole brain was acquired to provide detailed anatomy (G.E. Fast Spoiled Gradient Recalled Echo sequence, matrix size = 256 * 256, FOV = 256 mm^2^, 134 slices, slice thickness = 1 mm, no gap, total scan time = 2 min 56 s).

### fMRI analysis

Image time-series analysis was performed using BrainVoyager QX 2.4 (Brain Innovation, Maastricht, The Netherlands) (Goebel et al., 2006). The time-series were corrected for slice acquisition time, realigned with their corresponding T1 volumes, warped into standard space (Talairach and Tournoux, 1988), re-sampled into 3 mm isotropic voxels, motion-corrected using Levenberg-Marquarts's least square fit for six spatial parameters, highpass-filtered for removal of low frequency drifts, corrected voxelwise for linear drifts, and spatially smoothed using a 8-mm full-width at half-maximum Gaussian kernel. The general linear model (GLM) was computed from the 18 z-normalized volume time courses. For all stimuli of interest, i.e., rest and stimulation periods, box-car time courses with a value of one for the stimuli of interest and values of zero for the remaining time points were convolved with a theoretical hemodynamic response function (Boynton et al., 1996) and were entered as predictors into the design matrix of the study. Contrast analyses were based on random effects GLMs of the z-normalized volume time courses. Analyses of the stimulation periods (all groups combined) vs. rest periods as well as contrast analyses between groups were performed from the 18 subjects using a statistical threshold of q(FDR) <0.05 corrected for multiple comparisons.

### Statistical analysis

The ratings of intensity and valence were normally distributed, according to a Shapiro-Wilk test, but there was no equality of variances across the means of intensity of our three groups (Bartlett test). We performed a Kruskall-Wallis test to search for differences between our three groups using the means of intensity (subjects' ratings) and valence (norms from the IAPS) for each stimulus. When a difference was detected, we performed a Student test to search for differences between each group of stimuli.

SCRs were not normally distributed (Shapiro-Wilk test). We used non-parametric tests. The differences in SCRs across the three groups of stimuli were assessed using a Kruskall-Wallis test and when a difference was found, comparison between groups was made by a Welch test. The differences in SCRs across the two sessions were assessed using a Wilcoxon test. Tests were undertaken for frequency of SCRs, magnitude, and amplitude (microsiemens).

## Results

### Ratings of intensity

**Figure 1:**
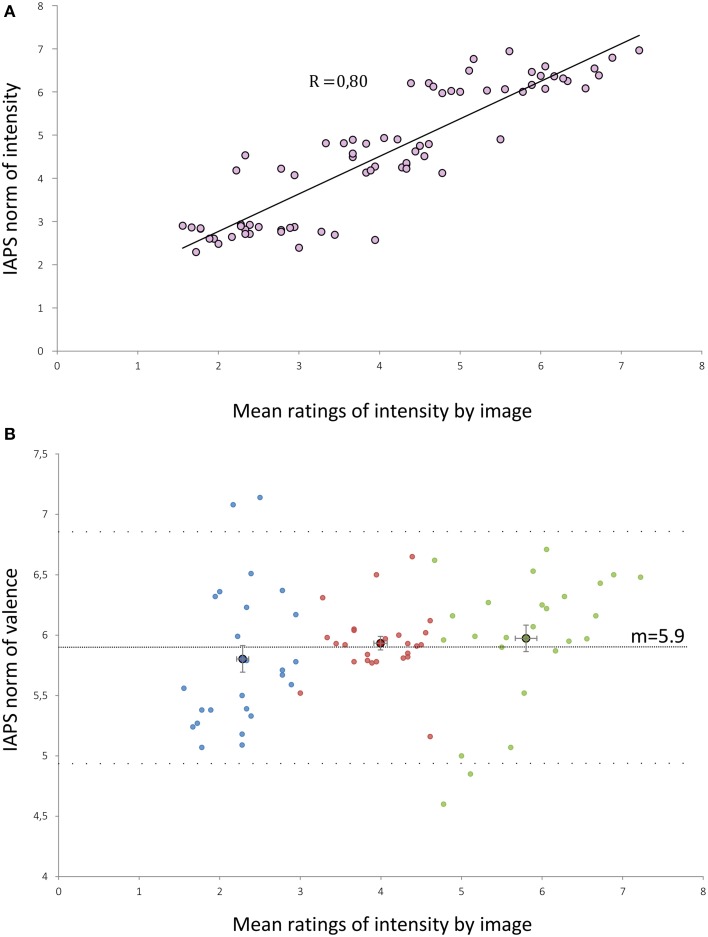
**(A)** Distribution of the mean intensity ratings by our subjects for each of the 75 selected IAPS pictures against the IAPS norms of intensity. R, correlation coefficient (Pearson test, *p* < 0.001). **(B)** Distribution of the mean intensity ratings by our subjects for each of the 75 selected IAPS pictures against the IAPS norms of valence. Dashed lines represent the mean valence (*m* = 5.9) and the mean ± 1.96 SD (4.94 and 6.87). Blue (or respectively red/green) points form the low (or respectively medium/high) intensity groups. Points with a black edge represent the means of the sub-groups. Error bars are the standard error of the mean.

Ratings made by our subjects were strongly correlated to IAPS norms of intensity (*r* = 0.80; *p* < 0.001) (Figure 1A). The ratings made by the subjects were slightly lower than the norms of the IAPS by 0.5 points (*p* < 0.001, paired *t*-test between IAPS ratings and mean ratings from our subjects). We built three groups of 25 visual stimuli according to the mean ratings of intensity performed by subjects: Group 1 (low intensity, *m* = 2.28), Group 2 (moderate intensity, *m* = 4), and Group 3 (high intensity, *m* = 5.8). Mean ratings of intensity were statistically different between each group of stimuli (Kruskall-Wallis *p* < 0.001). There was no significant difference between the mean valence of each group (Kruskall-Wallis, *p* = 0.13) (Figure 1B).

According to these three new groups, mean luminance for the group of low intensity (respectively medium intensity, high intensity) was 87.3 (respectively 86.4, 89.9). No statistical difference was observed between groups neither for this parameter, nor for the contrast.

### fMRI data

The global fMRI analysis comparing the viewing of stimuli and rest state showed activation in the right and left anterior cingulate gyri, right, and left superior medial frontal gyri, right, and left inferior frontal gyri, right, and left posterior orbital gyri, right, and left anterior part of insula, right, and left occipital gyri, right, and left thalami (medial thalamus and pulvinar), right and left colliculi, right, and left amygdalae, right, and left parahippocampal gyri, and the vermis. These areas are known to be involved in the processing of visual emotional information (Sabatinelli et al., 2011).

The fMRI analysis between the three groups of stimuli (according to subjects' intensity ratings) showed activations in the right and left amygdalae, the right orbital gyrus, the right pulvinar, the right, and left medial thalami, the anterior part of the insula, the right, and left colliculi, and the hypothalamus (see Tables 2, 3 for a full description).

**Table 2:** **Cerebral activations for the 18 healthy subjects for the 3 comparisons between the 3 levels of emotional intensity: Group 1, during viewing of low intensity stimuli; Group 2, during viewing of medium intensity stimuli; Group 3, during viewing of high intensity stimuli**.

**Group 3 >Group 1**	**Group 2 > Group 1**	**Group 3 > Group 2**
Left amygdala	×	×
Right amygdala	×	×
Right orbital gyrus	×	×
Right dorsolateral prefrontal cortex (inferior frontal gyrus)	×	Right dorsolateral prefrontal cortex (inferior frontal gyrus)
Right medial frontal gyrus	Right medial frontal gyrus	×
Left medial frontal gyrus	Left medial frontal gyrus	×
Right fusiform gyrus	Right fusiform gyrus	×
Left parahippocampal gyrus		
Right parahippocampal gyrus	×	Right parahippocampal gyrus
Right temporo-occipital junction	×	Right temporo-occipital junction
Left temporo-occipital junction	×	Left temporo-occipital junction
Left parieto-occipital junction	×	×
Right posterior cingulate	×	×
Left posterior cingulate	×	Left posterior cingulate
Left anterior insula		
Right and left colliculi	×	×
Right pulvinar	×	×
Left medial thalamus	×	Left medial thalamus
Right medial thalamus	×	×
Right hypothalamus	×	×
Vermis	×	×

*Analyses were based on random effects (RFX) GLMs of the z-normalized volume time courses using a statistical threshold of p < 0.05 corrected for FDR*.

**Table 3:** **Cerebral activations for the 18 healthy subjects when testing Group 3 (during viewing of high intensity stimuli) against Group 1 (during viewing of low intensity stimuli)**.

**Area (*p* < 0.001)**	**BA**	***x***	***y***	***z***	**Peak (max *t*-value)**	**Size (voxels)**
Left amygdala		−22	−8	−9	5.134	777
Right amygdala		17	−2	−12	5.130	750
Vermis		−4	−59	−43	6.081	923
Right fusiform gyrus	20	32	−41	−18	6.739	3139
Right orbital gyrus	47	29	13	−18	7.477	1898
Right temporo−occipital junction	37	38	−59	9	6.445	5821
Left temporo−occipital junction	37	−53	−71	3	6.943	3838
Right parahippocampal gyrus	28	23	−29	−9	4.910	1698
Left parahippocampal gyrus	28	−10	−35	−9	5.923	1907
Right colliculus		6	−32	−2	4.848	112
Left colliculus		−5	−35	−7	5.126	209
Right pulvinar		11	−32	3	5.291	1059
Left parieto−occipital junction	19	−16	−59	3	4.788	377
Right posterior cingulate	23	2	−56	18	5.473	1579
Left posterior cingulate	23	−2	−59	21	5.007	534
Left medial thalamus		−4	−14	−3	7.386	1259
Right medial thalamus		0	−14	−3	5.956	556
Right dorsolateral prefrontal cortex (inferior frontal gyrus)	9	38	10	27	6.032	1926
Left anterior insula		−34	28	0	4.119	68
Right hypothalamus		13	−2	−12	4.755	158
Left medial frontal gyrus	10	−1	55	9	4.766	979

*Analyses were based on random effects (RFX) GLMs of the z-normalized volume time courses using a statistical threshold of p < 0.05 corrected for FDR. Size (voxels), size of the cluster in number of connected voxels of 1 mm^3^; x, y, z, Talairach coordinates of the maximum peak. BA, Brodmann area*.

**Figure 2:**
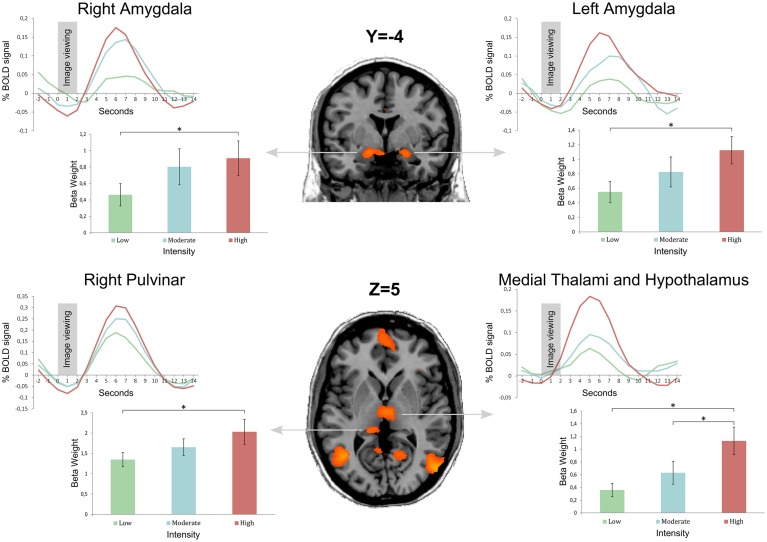
**Bold curves as well as mean beta weight calculated from 18 subjects by image group in four regions of interest: left and right amygdalae, right pulvinar, and the medial thalami and hypothalamus complex**. Group 1 is the image set with the lowest intensity, and Group 3 the image set with the highest intensity (Group 2 is an intermediate intensity image set). Regions of interest were obtained from the contrast Group 3 minus Group 1 after a statistical threshold of q(FDR) < 0.05 corrected for multiple comparisons. Error bars represent the standard error of the mean (± s.e.m.) * indicates a <0.05 *p*-value.

Regarding the main objective of the study, we found a stronger activation of the right and left amygdalae when subjects visualized stimuli of stronger intensity (Group 3) compared to stimuli from the group of lower intensity (Group 1). The left and right amygdalae were activated by viewing emotional stimuli, as demonstrated by the analysis of the differences of cerebral activation between no stimulation (black cross) and the viewing of pictures. However, amygdala activation was stronger when subjects viewed stimuli with strong intensity compared to stimuli with low intensity, with the same valence (neutral or positive). There was no significant difference with corrected statistics in intermediary comparison (between Groups 1 and 2 and between Groups 2 and 3) for the amygdalae. These results are illustrated on Figure 2 after extracting beta values for each condition in four regions of interest (left and right amygdalae, right pulvinar, and the medial thalami, and hypothalamus complex) defined from the contrast Group 3 minus Group 1.

### Skin conductance responses (SCRs)

#### First session, inside the scanner

**Figure 3:**
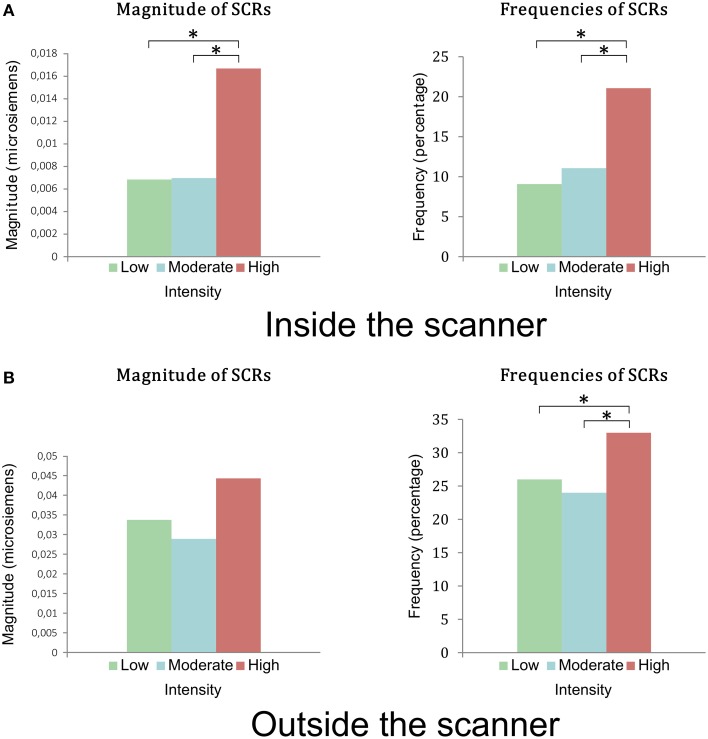
**(A)** Magnitudes and frequencies of the SCRs during viewing of stimuli in the first session inside the scanner. There was a statistical difference between groups of low and moderate intensity compared to the group of high intensity in terms of magnitude and frequency of SCRs. **(B)** Magnitudes and frequencies of the SCR during the viewing of the stimuli outside the scanner, in the second session. There was no statistical difference between the magnitudes of the 3 groups (*p* = 0.28). There was a statistical difference in frequencies of SCRs between groups of low and moderate intensity compared to the group of high intensity. *indicates a <0.005 *p*-value.

During the fMRI session, 16 subjects presented SCRs and 2 subjects (2 women) presented no SCRs. The magnitude and frequency of SCRs were greater for the stimuli in Group 3, rated by subjects as the most intense (*p* < 0.005) (Figure 3A). There was no difference in amplitude between the three groups of stimuli (*p* = 0.09).

#### Second session, 1 month later, outside the scanner

During the second phase, 17 subjects presented SCRs and 1 subject showed no SCRs (a woman who did not present any SCRs in the first phase). The frequency of SCRs for Group 3 was significantly higher than those of Groups 1 and 2 (*p* = 0.041) (Figure 3B). There was no significant difference in magnitude or amplitude in SCRs in the three groups of stimuli (*p* = 0.27 and *p* = 0.5 respectively).

#### Comparison of the session inside the scanner with the session outside the scanner

Frequency, magnitude, and amplitude of the SCRs were higher during the second pass, outside the MRI scanner, compared to the first pass in the MRI scanner (respectively: frequency = 14 vs. 27%; magnitude = 0.01 vs. 0.04; amplitude = 0.08 vs. 0.12; *p* < 0.0001).

Frequencies of electrodermal responses were significantly correlated between sessions 1 and 2 (*r* = 0.67; *p* = 0.002). Magnitudes of electrodermal responses were also significantly correlated between sessions 1 and 2 (*r* = 0.79; *p* < 0.001).

## Discussion

### Amygdala and emotional intensity

We have shown that the amygdala is sensitive to the emotional intensity of positive stimuli. This result is consistent with studies in primates (Belova et al., 2007) and some studies in humans (Phan et al., 2003, 2004; Sabatinelli et al., 2005; Cunningham and Kirkland, 2014). However, these studies often showed a bias linked to the use of stimuli varying both in intensity and valence within the same protocol. Indeed, if we consider that the amygdala is sensitive to both the size of intensity and valence, the valence effect may mask an intensity effect if it is stronger or dependent on a higher number of amygdaloid neurons. That is why we chose to use only positive emotional stimuli in this study, to avoid the bias linked to valence. As the valences are not different between our three groups of pictures, our result is not linked to the “valence” parameter. Studies in macaques have shown that there are different groups of amygdaloid neurons that do not encode the same information. Some amygdaloid neurons were activated depending on the intensity of positive stimuli, some depending on the intensity of negative stimuli and others depending on the intensity of the two types of stimuli. These neurons are involved in mechanisms independent of valence (Belova et al., 2007). Optogenetics in mice has shown that different amygdala nuclei belong to various functional and anatomical circuits (Lalumiere, 2014) involved in anxiety, fear-related behavior and regulating reinforcement and reward). Due to the limits of the resolution of functional MRI, it is not possible to accurately discriminate the different amygdaloid nuclei. To clarify the roles and networks to which they belong, further studies using other techniques, such as tractography, are needed (Solano-Castiella et al., 2010).

### Amygdala and lateralization

Our study did not reveal any specific amygdala lateralization. Various models of amygdala lateralization have been proposed, with more recent models showing that amygdala lateralization may be linked to the temporal dynamics of information processing (Wright et al., 2001). Activation of the amygdala found most frequently on the left rather than the right in some studies and meta-analyses (Murphy et al., 2003; Wager et al., 2003; Baas et al., 2004) may be related to a habituation or temporal dynamics effect. The right amygdala is involved in the initial and rapid detection of stimuli, processing data over a shorter duration of time, compared to the left amygdala that may more elaborately evaluate stimuli with a longer latency response (Gläscher and Adolphs, 2003; Sergerie et al., 2008). This is consistent with the conclusion of several meta-analyses that have shown no amygdala lateralization according to stimuli valence, or sex of the subjects (Murphy et al., 2003; Wager et al., 2003; Baas et al., 2004). Interaction between amygdala laterality and task, stimulus type or novelty may not exist either (Baas et al., 2004).

### Other activations associated with emotional intensity in this study

Our study showed activation of the medial thalamus and the posterior orbital and medial prefrontal cortices, parallel to the increase in the intensity of the stimuli. Concordantly with our study, a coding of emotional intensity by the medial thalamus and the medial prefrontal cortex was demonstrated (Anders et al., 2004). The amygdala is connected to the prefrontal cortex in neocortical circuits that incorporate emotional significance of stimuli and guide complex behavior. The amygdala sends pertinent, filtered sensory information to the prefrontal cortex (directly or via the mediodorsal nucleus of the thalamus) and the latter returns reinforcement or inhibition information to the amygdala after more cognitive processing of information. This is likely to be due to the reciprocal connections between the amygdala and the prefrontal cortex, sources of individual emotional experience specific to humans (Purves et al., 2005; Kim et al., 2011).

We have demonstrated that the right pulvinar and the left and right colliculi are sensitive to emotional intensity. Data for this circuit are controversial. For some, it may be responsible for the unconscious processing of visual emotional stimuli, while for others, its role, like that of the amygdala, may be to coordinate the neocortical circuits in the treatment of the relevance of visual emotional information (Buchsbaum et al., 2006; Pessoa and Adolphs, 2010; Sabatinelli et al., 2011).

Activation of the hypothalamus according to emotional intensity is consistent with amygdala activation. The latter sends projections to the hypothalamus and brainstem, allowing the expression of emotions through the modulation of autonomic and vegetative efferent motor systems. This was highlighted in this study by concomitantly monitoring the EDA, a marker of activation of the sympathetic system of the autonomic nervous system (Dawson et al., 2007).

### Validation of stimuli intensity scoring of the IAPS

Scoring the intensity of the IAPS photographs by subjects is similar to that provided by the IAPS. The average scoring of intensity by our subjects is slightly lower to that of the IAPS norms (minus 0.5 points), which is similar to scoring carried out by our neighboring countries (Switzerland, Germany; Bradley and Lang, 1994).

Thanks to the IAPS norms, we were able to build a set of stimuli sensitive to intensity, devoid of bias relating to valence, and validated in our population of subjects.

### Electrodermal activity and emotional intensity felt

In both sessions (inside and outside the MRI scanner), frequency of SCRs was greater for more intense stimuli (Group 3). Magnitude was only significantly higher for the more intense stimuli in the first session, in the MRI scanner. In contrast to data from other studies (Dawson et al., 2007) that have demonstrated an increase in the amplitude of SCRs with emotional intensity, we did not demonstrate any significant variation in the amplitude of SCRs according to intensity. Post-processing of the recording and MRI scan pass are not concerned because there is no relationship between the amplitude of SCRs and emotional intensity during the 2 phases (inside and outside the MRI scanner, not requiring post-processing).

We obtained identical statistical results in both sessions on the relationship between stimuli intensity and amplitude of SCRs (no link during both sessions), and the relationship between stimuli intensity and frequency of SCRs (increased for Group 3 in the 2 sessions). The only difference in result between sessions is magnitude. The magnitude of SCRs is related to stimuli intensity only during the first phase, in the MRI scanner. There is a non-significant tendency during the second phase, outside the MRI scanner. One can draw several hypotheses to explain this difference. It is, first of all, a matter of calculation of magnitude, involving both the concept of response frequency and of amplitude. As amplitude was not sensitive to stimuli intensity in our study, it is likely that it is the frequency that makes variations in magnitude significant. Furthermore, it is also possible that the lack of significance of magnitude in the second session is related to a habituation effect to stimuli. Intra-subject stability of SCRs has been demonstrated 1 year later (Schell et al., 2002) but our second phase took place 1 month after the first. Finally the significance of the relationship between magnitude of SCRs and stimuli intensity in the MRI scanner might be linked to an effect specific to undergoing an MRI. The environment of the MR scanner, noise, lying down, the stress of the examination or its cramped nature could make the subject more stressed and therefore more responsive to more intense stimuli, or less responsive to less intense stimuli. This issue is important because it raises the question of comparability of emotional states inside and outside the MRI scanner. Ambient temperature, a factor known to modify SCRs (Dawson et al., 2007), is not a confounding factor because it was controlled during the two sessions.

The values of the magnitudes, amplitudes, and frequencies of SCRs that we recorded in the MRI scanner are lower than those recorded outside the MRI scanner (Figure 3). Two factors may be responsible for this difference. First, there could have been a loss of signal due to post-processing of the EDA recording necessary when performing in the MRI. Despite MRI compatibility of the equipment used in this study, the EDA recording was artifacted by the functional MRI sequences and therefore required post-processing by selective removal of high frequencies and smoothing. However, these treatments are known to lose information, both in terms of the frequency of SCRs and in terms of their amplitude. The use of fiber optic cables (Lagopoulos et al., 2005) would avoid these artifacts and thus the loss of data linked to post-processing (Shastri et al., 2001). Undergoing an MRI scan by the subject is another factor that may explain the differences between the two sessions. The MRI might, via the stress that it can cause, block spontaneous emotional reactions. Our study cannot answer these questions. However, it can dispute the authenticity of emotional responses in the MRI scanner.

## Conclusion

These results highlight the sensitivity of the amygdala to variations in the intensity of positive emotions, and in conjunction with results in the literature on negative emotions, show the role of the amygdala in the perception of emotional intensity.

However, the role of the amygdala has been identified in other areas, such as the behavior of consciousness, attentional mobilization (Pribram and McGuinness, 1975), alertness, as well as the treatment of ambiguity of stimuli (Pessoa and Adolphs, 2010). Furthermore, the anatomical location and multiple connections of the amygdala mean that it may be a center for data processing, a role more than that of a simple “danger detector” (Kim et al., 2011). The amygdala may be an early detector of the relevance of stimuli for the individual (Sander et al., 2003). Its activation in the perception of the intensity of emotional stimuli may be only a facet of its overall role in general sensory perception.

The study of emotions in healthy subjects will eventually lead to a better understanding of dysfunctions in the pathology of emotions. The amygdala has been studied in several psychiatric pathologies involving emotional disorders such as anxiety disorders and schizophrenia. However, as its roles are multiple, underpinned by its many connections with cortical and subcortical structures, the pathophysiology of the amygdala is not limited to emotional disorders themselves but in fact extends to areas such as memory, attention, decision-making, and cognition.

### Conflict of interest statement

The authors declare that the research was conducted in the absence of any commercial or financial relationships that could be construed as a potential conflict of interest.

## Abbreviations

EDA: electrodermal system
IAPS: International Affective Picture System
SCR: skin conductance response.
